# IFI35 Promotes Renal Cancer Progression by Inhibiting pSTAT1/pSTAT6-Dependent Autophagy

**DOI:** 10.3390/cancers14122861

**Published:** 2022-06-09

**Authors:** Dafei Chai, Shang Yuchen Shi, Navid Sobhani, Jiage Ding, Zichun Zhang, Nan Jiang, Gang Wang, Minle Li, Hailong Li, Junnian Zheng, Jin Bai

**Affiliations:** 1 Cancer Institute, Xuzhou Medical University, Xuzhou 221002, China; dafei.chai@bcm.edu (D.C.); dmaity@uncc.edu (J.D.); dmaity2@uncc.edu (Z.Z.); dmaity3@uncc.edu (N.J.); wangg@xzhmu.edu.cn (G.W.); liminle123@163.com (M.L.); 2 Center of Clinical Oncology, Affiliated Hospital of Xuzhou Medical University, Xuzhou 221002, China; 3 Jiangsu Center for the Collaboration and Innovation of Cancer Biotherapy, Cancer Institute, Xuzhou Medical University, Xuzhou 221002, China; 4 Department of Medicine, Section of Epidemiology and Population Sciences, Baylor College of Medicine, Houston, TX 77030, USA; navid.sobhani@bcm.edu; 5 Department of Stereotactic Radiotherapy, The Second Affiliated Hospital of Xuzhou Medical University, Xuzhou 221006, China; ssyc0725@163.com; 6 Department of Urology, Affiliated Hospital of Xuzhou Medical University, Xuzhou 221002, China; justinlee719@hotmail.com

**Keywords:** RCC, renal cancer, IFI35, therapeutic target, diagnostic biomarker, autophagy

## Abstract

**Simple Summary:**

Interferon-induced protein 35 (IFI35) plays an important role in virus-related immune inflammatory responses. However, the biological significance and function of IFI35 in cancer is still not well understood. Our goal is to explore whether IFI35 could be used as tumor marker or a therapeutic target for renal cell cancer (RCC). Our results showed that IFI35 expression was significantly increased in 200 RCC specimens, and its expression was negatively associated with poor overall or disease-specific 5-year patients’ survival. Next, we explored the mechanism and function of IFI35 in RCC. The results demonstrated that knockdown of IFI35 could inhibit the proliferation and invasion of renal cancer cells by enhancing the STAT1/STAT6 phosphorylation (pSTAT1/pSTAT6)-dependent autophagy. Likewise, the knockdown of IFI35 also suppressed the tumor growth or lung metastasis of renal cancer by enhancing the induction of autophagy. This study aims to provide a theoretical basis for IFI35 as a potential diagnosis and therapeutic target for RCC or other cancers with high levels of IFI35.

**Abstract:**

Interferon-induced protein 35 (IFI35), is currently acknowledged to govern the virus-related immune inflammatory responses. However, the biological significance and function of IFI35 in renal cell cancer (RCC) is still not well understood. Here, IFI35 expression and function were investigated in RCC tissues, renal cancer cells, and animal models. The results showed that IFI35 expression was significantly increased in 200 specimens of RCC patients. We found that higher IFI35 levels were significantly correlated with poor RCC prognosis. In human cell lines, the knockdown of IFI35 suppressed the malignant behavior of renal cancer cells. Similarly, the IFI35 knockdown resulted in significant inhibition of tumor progression in the subcutaneous or lung metastasis mouse model. Furthermore, the knockdown of IFI35 promoted the induction of autophagy by enhancing the autophagy-related gene expression (LC3-II, Beclin-1, and ATG-5). Additionally, blockade of STAT1/STAT6 phosphorylation (pSTAT1/pSTAT6) abrogated the induced autophagy by IFI35 knockdown in renal cancer cells. The autophagy inhibitor 3-MA also abolished the prevention of tumor growth by deleting IFI35 in renal cancer models. The above results suggest that the knockdown of IFI35 suppressed tumor progression of renal cancer by pSTAT1/pSTAT6-dependent autophagy. Our research revealed that IFI35 may serve as a potential diagnosis and therapeutic target for RCC.

## 1. Introduction

Renal cell carcinoma (RCC) is the most common type and accounts for more than 90% of cancers in the kidney [1,2]. The clear cell RCC (ccRCC), papillary RCC (pRCC), and chromophobe RCC (chRCC) are the three most common histologic subtypes [3,4]. RCC shows a poor prognosis and results in high mortality rate, which is attributed to the refractory to traditional treatment [5,6]. Patients with metastatic RCC show rapid progression and worse survival in contrast to non-metastatic patients [7]. In recent years, great progress (surgical resection, immunotherapy, or targeted agents) has been made in the treatment of RCC to improve patient survival [8,9]. Nevertheless, RCC patients still present severely poor prognosis and high recurrence post-treatment in most cases [10]. Therefore, the mechanism of tumor progression requires further research to determine new therapeutic targets for RCC therapy.

Autophagy is a critical regulator for the maintenance of cellular homeostasis that is involved in the pathogenesis of RCC [11,12]. The study shows that autophagic gene polymorphisms are associated with progression-free survival (PFS) of ccRCC patients treated with pazopanib (while ATG16L2 shows a positive correlation, ATG4A, ATG4C and ATG5 show a negative correlation) [13]. Moreover, the activation of autophagy can be up-regulated by the inactivation of the phosphoinositide 3-kinase (PI3K)/protein kinase B (AKT)/mammalian target of rapamycin (mTOR) pathway in renal cancer or other tumors [14]. Furthermore, it has been reported that absent in melanoma 2 (AIM2)-induced autophagy is a potential cell inhibited mechanism of metastatic RCC, and this autophagy inhibition could block its therapeutic effect [15]. These studies indicate that the regulation and function of autophagy are closely correlated with tumor cell homeostasis and tumor pathogenesis and targeting therapy. Therefore, it stands to reason that the regulation of autophagy has great potential as a treatment for renal cancer. 

IFN-induced protein 35 (IFI35), is a 35 kDa protein that was first identified by screening of cDNA libraries of HeLa cells stimulated with IFN-γ [14]. IFI35 has been proposed to play a key role in modulating inflammatory immune response during antivirus-related immune processes [16,17]. The function of IFI35 also participates in the regulation of other inflammation-related diseases such as sepsis, liver injury, rheumatoid arthritis, and systemic lupus erythematosus (SLE) [18,19,20,21]. Furthermore, the expression of IFI35 was involved in cell proliferation, apoptosis, and migration by inhibiting the nuclear factor-kappa B (NF-κB) pathway in fibroblasts, macrophages, epithelial cells, and HeLa cells [22,23]. Therefore, the function of IFI35 has been well elucidated in cell homeostasis and innate immunity, but its biological and pathological roles in cancer are not well characterized. Therefore, whether IFI35 regulated autophagy-mediating homeostasis of renal cancer cells needs to be further studied. In this study, we first analyzed the prognostic significance of IFI35 expression in RCC and found that increased expression of IFI35 in RCC specimens contributed to disease progression. Furthermore, the role and mechanism of IFI35 in renal cancer were explored by the cell and animal models. Knockdown of IFI35 could inhibit the proliferation and invasion of renal cancer cells by enhancing the STAT1/STAT6 phosphorylation (pSTAT1/pSTAT6)-dependent autophagy. Our results indicated that knockdown of IFI35 could inhibit the tumor progression by enhancing autophagy, indicating that IFI35 could be a potential therapeutic target since it is highly expressed in cancer.

## 2. Materials and Methods

### 2.1. Patient Specimens and Ethics Statement

A total of 200 cases of tissue specimens from RCC patients who accepted curative surgery without prior treatment or postoperative adjuvant therapy were collected from the Department of Pathology of the Affiliated Hospital of Xuzhou Medical University between years 2005 and 2008. A total of 25 cases of normal renal tissues (NRT) were used as control. RCC patients’ clinicopathologic information (sex, age, tumor size, etc.,) were obtained from the archive of the pathology department and confirmed by the medical record of the hospital. The tissue microarray (TMA) was constructed by using tissue specimens at the National Engineering Center for Biochip (Shanghai, China) by a contract service approved by the local ethical committee. The diameter of every array dot was 1.5 mm, and each dot represented a tissue from one individual specimen. This study involved human subjects; hence it was carried out after obtaining written informed consent from all patients. All experiments were performed under a protocol approved by the institutional review boards of Affiliated Hospital of Xuzhou Medical University. 

### 2.2. Immunohistochemistry (IHC) Staining

Deparaffin and antigen retrieval were performed in TMA slides. Subsequently, the slides were blocked with 10% BSA and incubated with primary anti-human IFI35 mAb (clone: B-1; Santa Cruz Biotechnology, Dallas, TX, USA) overnight at 4 °C. The sections were incubated with a biotin-labeled goat anti-mouse secondary antibody (Zhongshan Biotech, Beijing, China) at 37 °C for 30 min. After washing with PBS, the sections were incubated with streptavidin-peroxidase (Zhongshan Biotech). Finally, the 3, 3′-diaminobenzidine (DAB) detection kit (Zhongshan Biotech) was used for staining and hematoxylin was used for counterstaining the nuclei. Immunostaining evaluation was performed as described in our previous material and methods [24]. Briefly, the evaluation of IFI35 staining was simultaneously done blindly by two pathologists using a microscope with multiple viewing, therefore, blinded to each other’s results. The intensity staining of IFI35 was conducted with the following 4 parameters: 0 = negative, 1 = weak, 2 = moderate, 3 = strong. IFI35 positive staining percentages were also scored into 4 categories: 1 (0–25%), 2 (26–50%), 3 (51–75%), and 4 (76–100%). Evaluation of IFI35 staining was conducted using immune reactive scores (IRS), which was calculated through the multiplication of the staining intensity scores with the positive cell percentages. Through the calculation of IRS, IFI35 staining pattern could be defined in the following four categories: negative (IRS: 0), weak (IRS: 1–4), moderate (IRS: 6–8), and strong (IRS: 9–12).

### 2.3. Western Blotting 

Protein samples (10 μg/well) were separated by electrophoresis in 12% SDS-PAGE gel, and then the gel was transferred to polyvinylidene fluoride (PVDF) membrane (Millipore) at 100 V for 2 h. The membrane was blocked with 5% de-fatted milk and incubated with primary anti-human antibodies as follows: IFI35 (clone: B-1; Santa Cruz Biotechnology), microtubule-associated light chain protein 3 (LC3) including LC-3-I and LC3-II (clone: D3U4C; Cell Signaling Technology, Danvers, MA, USA), ATG5 (clone: D5G3; Cell Signaling Technology), Beclin-1 (clone: D40C5; Cell Signaling Technology), or β-actin (clone 13E5; Cell Signaling Technology), followed by goat anti-mouse or goat anti-rabbit IgG-HRP (Cell Signaling Technology). The signals were developed with the SuperSignal West Pico Chemiluminescent Substrate (Thermo Scientific, Waltham, MA, USA). Bands were quantified using ImageQuant software (Tanon, Shanghai, China).

### 2.4. Cell Culture and Stable Cell Line Selection

Normal renal tubular epithelial cell line (HK-2) and human renal cancer cell lines (ACHN, Ketr-3, and 786-O) were purchased from the Chinese Academy of Sciences (Shanghai, China). The human embryonic kidney cell line (HEK-293T) waspurchased from American Type Culture Collection (ATCC, Manassas, VA, USA). These cells were cultured in Dulbecco’s modified Eagle medium (DMEM, Gibco, Thermo Fisher Scientific) supplemented with 10% fetal bovine serum (ExCell Bio, Shanghai, China), 100 units/Ml penicillin (Sango Biotech, Shanghai, China), and 100 μg/mL streptomycin (Sango Biotech) at 5% CO_2_ atmosphere at 37 °C. All cells were routinely tested for mycoplasma contamination and authenticated by short tandem repeat profiling analysis as well as passaged for less than 6 weeks before renewal from frozen, early passage stocks. 

The pPLK-shRNA-IFI35 plasmid (shRNA-1 target sequence: 5′- CTGGTGCTCAACATTCCTGAT -3′; shRNA-2 target sequence: 5′- GCTGGACAAGCTAGAGATCTT -3′) and negative control shRNA (sequence: GTTCTCCGAACGTGTCACGTT) were bought from Public Protein/Plasmid Library (PPL, Nanjing, China). For stable cell lines of IFI35 knockdown, Ketr-3 and 786-O cells were plated into 6-well plates (Corning, Corning, NY, USA) and infected with IFI35 shRNA (shRNA-1 or shRNA-2) or control shRNA (shRNA-Ctrl) lentivirus, which had been developed by co-transfecting with psPAX2 and pMD2.G packaging plasmids in 293T cells. Forty-eight hours after infection, the cells were split and stable transfectants were selected by puromycin (Solarbio, Beijing, China). The selected stable cells were expanded and used for the following studies. In the autophagy inhibitor study, cells were pretreated with 3 mmol/L 3-Methyladenine (3-MA, Sigma-Aldrich, St. Louis, MO, USA).

### 2.5. Cell Proliferation Assay

The xCELLigence RTCA SP instrument (ACEA Biosciences, San Diego, CA, USA) was used to measure cell proliferation. Briefly, the background impedance was measured by adding 50 μL of cell culture media to each well of 2 × 8-well E-Plates (ACEA Biosciences) and displayed as the cell index. Subsequently, cells were seeded onto E-Plates at a density of 1 × 10^4^ cells/well in a volume of 150 μL and allowed to passively adhere to the electrode surface. The E-Plates were maintained at ambient temperature in a laminar flow hood for 30 min and then transferred to the RTCAMP instrument in a cell culture incubator. Data recording was initiated immediately at 15-min intervals for the entire duration of the experiment. Data acquisition was resumed to monitor the cells based on the viability of attached cells as reflected by cell index values.

### 2.6. Cell Apoptosis Analysis

Cell apoptosis was determined by the Annexin V-FITC/PI apoptosis detection kit (BD Biosciences, San Jose, CA, USA) according to the manufacturer’s instructions. Briefly, cells were harvested using 0.05% trypsin, and washed with a staining buffer. The cells were then stained with FITC-labeled Annexin V and PI for 5 min at 4 °C and subjected to flow cytometric analysis utilizing a FACSCanto II flow cytometer (BD Biosciences). The percentage of distribution of cell apoptosis was calculated by FlowJo software (Tree Star, Ashland, OR, USA).

### 2.7. Cell Invasion Assay

For invasion assay, 30 μL of matrigel basement membrane matrix (BD Biosciences) diluted with serum-free medium (diluted 1:8) was added to the upper compartment of the 24-well trans-well culture chamber (8 μm pore size; Corning Costar). About 6 × 10^4^ cells suspended in 200 μL of serum-free medium were seeded on the upper compartment, and 600 μL of complete medium was added to the lower compartment. Twenty-four hours after incubation at 37 °C, cells were fixed with methanol. The invaded cells on the lower side of the filter were stained with crystal violet and counted.

### 2.8. Analysis of Autophagy by Fluorescence Microscope

GFP-RFP-LC3 fusion protein (two-color fluorescent) was used to trace the process of autophagy. RFP-GFP-LC3 emitted both green and red fluorescence signals when the protein localizes to autophagosomes (yellow signals in merged pictures) in autophagy induction process. Renal cancer cells were seeded onto glass cover slides (1 × 1.5 cm) in 6-well plates (Corning) overnight. The cells were transfected with tandem GFP-RFP-LC3 adenovirus (Hanbio, Shanghai, China) according to the manufacturer’s instruction. Cells were incubated for 24 h, washed with PBS, fixed with 4% paraformaldehyde, stained with DAPI, and coverslipped. Confocal images were obtained using an Olympus FV1000 Laser Scanning confocal fluorescence microscope (Tokyo, Japan). Autophagosomes displaying both green and red fluorescence were evaluated by LC3 puncta number per cell quantified using ImageJ software.

### 2.9. Flow Cytometry Analysis

IFI35 expression was detected in HK-2, ACHN, Ketr-3, and 786-O cell lines. pSTAT1 or pSTAT6 expression was examined in Ketr-3 and 786-O cells transfected with IFI35 shRNA and control shRNA in the presence or absence of the signaling inhibitor Fludarabine (5 μmol/L; MedChem Express, Monmouth Junction, NJ, USA) or AS1517499 (100 nmol/L; MedChem Express) for 24 h. For intracellular staining, cells were washed and fixed with fixation buffer (BD Biosciences) for 20 min at 4 °C. The fixed cells were permeabilized with permeabilization solution (BD Biosciences) at room temperature for 30 min. Cells were incubated with PE-anti-human pSTAT1 (BioLegend, San Diego, CA, USA), PE-anti-human pSTAT6 (BioLegend) in PBS containing 2% FBS for overnight at 4 °C. Isotype-matched anti-human IgG served as a control. Subsequently, the labelled cells were analyzed using a BD FACSCanto II flow cytometer with FACSDiva software (BD Biosciences).

### 2.10. Animal Models

Female BALB/c nude mice (6- to 8-weeks of age) were purchased from Beijing Vital River Laboratory Animal Technology Co., Ltd. (Beijing, China) and housed in a specific pathogen-free room under controlled temperature and humidity. Animal care and all experimental procedures were carried out in strict accordance with the Guide for the Care and Use of Medical Laboratory Animals. All animal experimental protocols were approved by the guidelines of Xuzhou Medical University Laboratory Animal Ethical Committee.

For the subcutaneous tumor model, mice were randomly divided into four groups (each group contained five mice). 786-O-shRNA cells or control cells were washed twice with PBS and resuspended in a mixture of serum-free medium and matrigel basement membrane matrix with a 1:1 volume ratio. Each mouse was inoculated over the flank subcutaneously with 2 × 10^6^ cells in a total volume of 100 μL. Tumors were monitored weekly for 45 days by measuring tumor size using a caliper. The tumor volume was calculated using the following formula: V (mm^3^) = (length × width^2^)/2. The body weights of the mice were measured every three days. When the mean tumor volume had reached 50 mm^3^, mice were received intraperitoneal 24 mg/kg 3-MA dissolved in 100 μL PBS once every 5 d at a total of five times until reaching the endpoint.

For the lung metastasis model, mice were randomly divided into three groups (each group *n* = 5) and intravenously injected with 4 × 10^6^ 786-O-shRNA cells/mouse or control cells in the tail vein at day 0. On Day 56 after tumor inoculation, mice were sacrificed and lungs were removed, and the metastatic nodules were quantified.

### 2.11. Pathological Analysis

For routine histological analysis, lung tissues of mice were surgically resected and fixed in 4% paraformaldehyde (Sigma-Aldrich), embedded in paraffin, and cut into 4 μm sections. H&E staining was performed according to the manufacturer’s instructions. Briefly, sections were deparaffinized and stained with adequate hematoxylin for 5 min. The slide was rinsed twice with distilled water to remove excess stain. Then, the tissue section was covered with adequate bluing reagent to completely cover, and then incubated for 10–15 s. After rinsing the slides with distilled water, each slide was dipped in different concentrations of alcohol. The slide was cleared and mounted in synthetic resin. The H&E sections were evaluated by two pathologists blinded to the treatment or control groups and each other’s results. Pictures were acquired with a microscope (Nikon, Tokyo, Japan) equipped with photo software. 

### 2.12. Statistical Analysis

Statistical analysis was performed with SPSS 16.0 software or GraphPad Prism software. The association between the IFI35 staining and the clinicopathologic parameters of RCC patients was assessed by Fisher’s exact two-sided tests. The Kaplan–Meier method and log-rank test were used to evaluate the correlation between IFI35 expression and patient survival. Data were presented as means and standard deviations (means ± SD) and statistically analyzed with two-tailed independent Student’s *t*-test (two groups) or one-way analysis of variance (ANOVA) (>two groups). The level of statistical significance was set at * *p* < 0.05, ** *p* < 0.01, and *** *p* < 0.001. 

## 3. Results

### 3.1. Increased Expression of IFI35 Is Correlated with Poor Prognosis in RCC

**Figure 1:**
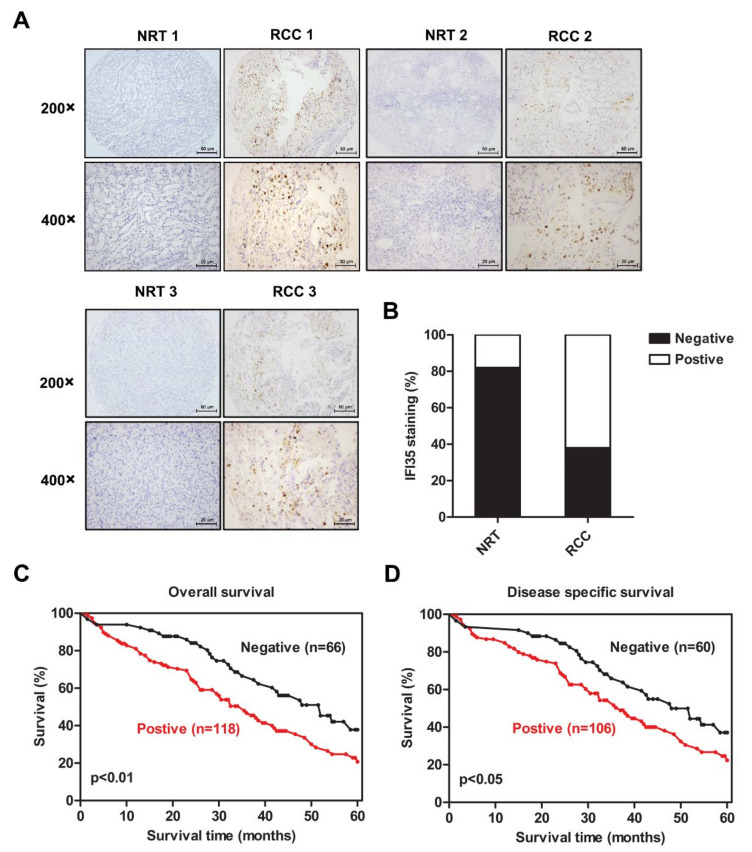
Higher IFI35 expression level was correlated with poor prognosis in RCC patients. (**A**). Represent immunohistochemical staining of IFI35 in RCC (renal cell carcinoma tissues) and NRT (normal renal tissues), top panel, ×200 magnification, scale bar: 50 µm, bottom panel, ×400 magnification, scale bar: 20 µm. (**B**). The percentages of IFI35 negative or positive expression assessed by the staining scores in (**A**) are shown in (**B**); the IHC staining data from 25 NRT and 200 RCC were available. (**C**). IFI35 expression correlates with a poorer overall survival (*p* = 0.0066). (**D**). The correlation analysis performed between IFI35 expression and disease-specific 5-year survival (*p* = 0.0325) is shown in this panel. Data are shown as means ± SD.

In order to determine the function of IFI35 in the pathogenesis of RCC, IFI35 expression was first detected in tumor tissues from TMA slides of patients. As shown in Figure 1A and Figure S1, IHC staining results showed that IFI35 expression was higher in RCC tissues than NRT. The staining score analysis of 200 biopsies suggested that the percentages of high IFI35 expression (positive) and low IFI35 expression (negative) were 62% (124/200) and 38% (76/200), respectively (Figure 1B). Table 1 summarized the clinicopathologic features of the RCC patients. Notably, increased expression of IFI35 was associated with poor overall (*p* < 0.05) or disease-specific 5-year patients’ survival (*p* < 0.05) (Figure 1C,D). These results indicated that high expression of IFI35 correlated with poor prognosis in RCC patients.

### 3.2. Knockdown of IFI35 Inhibits Cell Proliferation and Invasion and Promotes Cell Apoptosis in Renal Cancer Cells

**Figure 2:**
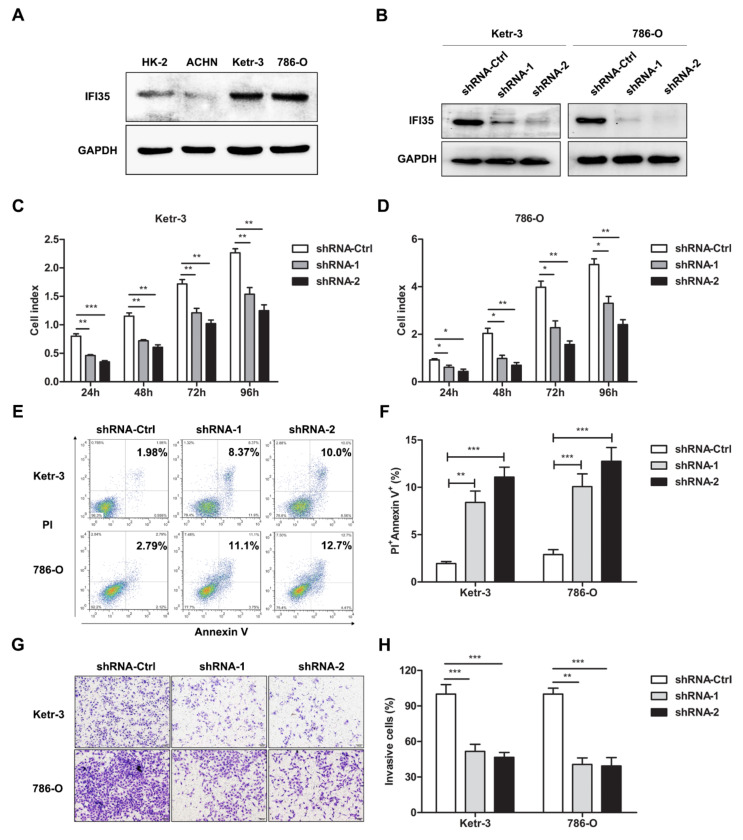
Knockdown of IFI35 inhibited cell proliferation and invasion and promoted cell apoptosis in renal cancer cells. (**A**) IFI35 expression was detected by Western blot in HK-2, ACHN, Ketr-3, and 786-O cell lines. (**B**) Forty-eight hours after IFI35-shRNA transfection, IFI35 expression was evaluated by Western blot in IFI35 shRNA-treated Ketr-3 or 786-O cells. (**C**,**D**) The proliferation assay conducted by RTCA was performed after shRNA-treated in Ketr-3 and 786-O. (**E**) The cell apoptosis was detected by flow cytometry in shRNA or shRNA-Ctrl-treated Ketr-3 and 786-O. (**F**) The percentages of apoptotic cells are represented in (**E**). (**G**,**H**) The invasion of Ketr-3 and 786-O cells was detected in the shRNA-treated group or shRNA-Ctrl-treated group. Data are from one representative experiment of three performed and presented as the mean ± SD, cell samples, *n* ≥ 3. Statistical significance was set at * *p* < 0.05, ** *p* < 0.01, and *** *p* < 0.001. Original blots see Supplementary File S1.

IFI35 expression was also detected in renal cancer cells by Western blot. As shown in Figure 2A, IFI35 expression increased in Ketr-3 or 786-O cells, while it was lower in ACHN cells compared to HK-2 cells. To investigate the role of IFI35 in the tumor progression of renal cancer, IFI35 was knocked down in renal cancer cells by lentivirus-mediated shRNA. Western blot results demonstrated that IFI35 expression was significantly reduced in IFI35 shRNA-treated Ketr-3 or 786-O cells, indicating that IFI35 expression was effectively suppressed by shRNA in renal cancer cells (Figure 2B). Through RTCA assay, cell proliferation was remarkably decreased in IFI-35 shRNA-treated renal cancer cells (Figure 2C,D). Moreover, increased cell apoptosis was also detected in the IFI35 shRNA-treated group (Figure 2E,F). Cell invasion was performed by trans-well assays in vitro, the results showed that cell invasive percentages of Ketr-3 or 786-O cells were markedly decreased in the IFI35 shRNA-treated group compared to the shRNA-Ctrl-treated group (Figure 2G,H). These results indicated that the knockdown of IFI35 in renal cancer could inhibit cell proliferation and invasion, as well as enhance cell apoptosis.

### 3.3. IFI35 Knockdown Suppresses the Tumor Growth or Metastasis in Subcutaneous or Lung Metastasis Models of Renal Cancer

**Figure 3:**
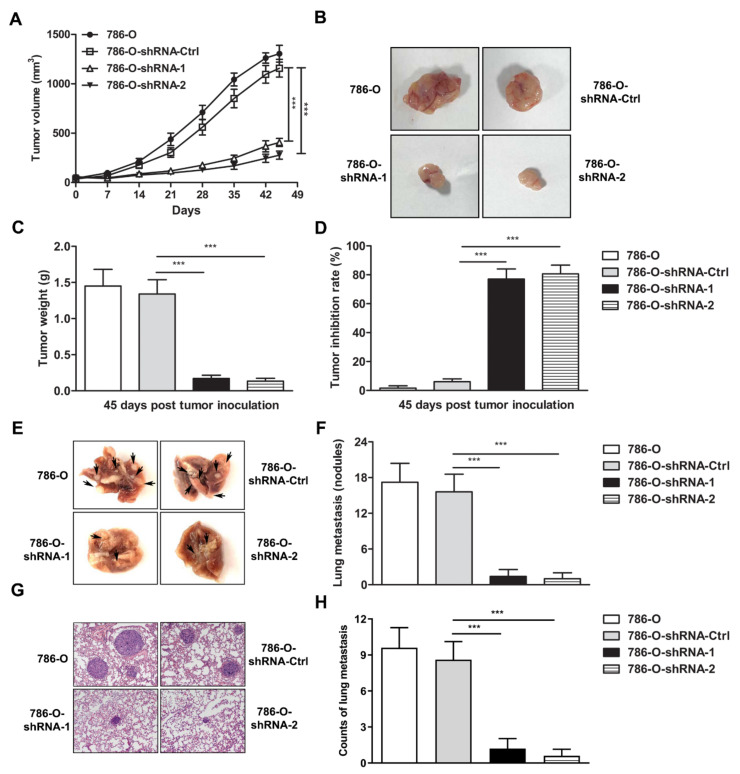
Inhibition of tumor growth of 786-O cell-xenografted nude mice by IFI35 knockdown. The nude mice were inoculated subcutaneously with shRNA-IFI35 or shRNA-Ctrl-treated 786-O cells. (**A**) Tumor progression of 786-O cell-xenograft was measured once every 7 days from day 0 to day 45. (**B**) Forty-five days after tumor inoculation, images of tumor excised from mice treated with shRNA or control shRNA were represented. (**C**) Tumor weight was illustrated. (**D**) Tumor inhibition rate was graphed. (**E**) The present images constituted lung tumors excised from tumor-bearing mice. (**F**) The numbers of metastatic nodules were quantified in the lung tissues. (**G**) H&E staining for lung tissues is presented (×200 magnification). (**H**) Lung metastasis counts were analyzed for comparisons among the four groups. The experiments were performed with five mice per group. Each experiment was performed independently at least three times and the results of one representative experiment are shown. Data are means ± SD, *** *p* <0.001.

To evaluate the anti-tumor effect of IFI35 knockdown in renal cancer models, the subcutaneous xenograft model and lung metastasis model were established in nude mice with 786-O-shRNA cells. In the subcutaneous model, tumor growth was inhibited in the 786-O-shRNA group compared with the 786-O-shRNA-Ctrl group at different time points. On day 45 after tumor inoculation, the 786-O-shRNA group had significantly suppressed tumor growth when compared with the control group (Figure 3A). Accordingly, the volume and weight of tumors were also decreased in the 786-O-shRNA group (Figure 3B,C). Moreover, knockdown of IFI35 inhibited tumor growth rate significantly in the IFI35 knockdown group vs the control group (Figure 3D). These data implied that the knockdown of IFI35 could effectively prevent the aggravation of tumor in the renal cancer cell-xenograft model. 

In the lung metastasis model, mice were sacrificed. The lungs were removed on day 56 after tumor inoculation, and the visible metastases were counted. A dramatically reduced number of lung metastases were observed in the 786-O-shRNA group compared with the control group (Figure 3E,F). The protective efficacy of IFI35 knockdown was further confirmed by H&E staining of lung tissues in the lung metastasis model (Figure 3G,H). These results indicated that knockdown of IFI35 also suppresses tumor lung metastasis of renal cancer.

### 3.4. Knockdown of IFI35 Enhances pSTAT1/pSTAT6 Dependent Autophagy in Renal Cancer Cells

**Figure 4:**
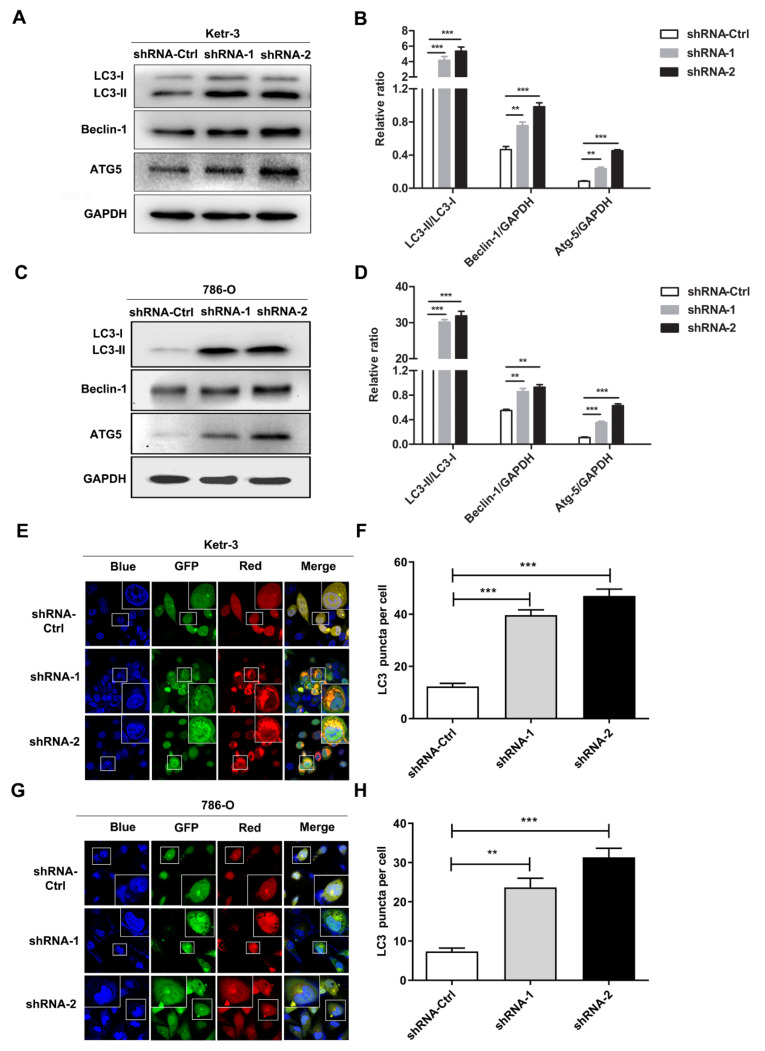
Knockdown of IFI35 enhanced autophagy induction in renal cancer cells. (**A**,**C**) The levels of LC3-I, LC3-II, Beclin-1, and ATG5 were measured by Western blot in IFI35 shRNA or shRNA-Ctrl-treated Ketr-3 and 786-O cell lines. (**B**,**D**) The statistical analysis of the band intensity represents a densitometric estimation of each band normalized by GAPDH in (**A**,**C**). (**E**,**G**) IFI-35 shRNA or shRNA-Ctrl-treated renal cancer cells were transfected with GFP-RFP-LC3 adenovirus and cultured for 24 h. Cells were fixed, stained with DAPI, and imaged with a 63× oil-immersion objective lens, scale bar: 20 µm. (**F**,**H**) LC3 puncta number per cell in (**E**,**G**) was quantified. Data are from one representative experiment of three performed and presented as the mean ± SD, cell samples, *n* ≥ 3. Statistical significance was set at ** *p* < 0.01, and *** *p* < 0.001. Original blots see Supplementary File S1.

Autophagy activation can be considered a tumor-suppressing mechanism for inhibiting tumor growth [25,26]. To investigate whether the knockdown of IFI35 regulated tumor progression by autophagy in RCC, we first detected the induction of autophagy in IFI35 shRNA-treated renal cancer cells. Compared with the control group, the IFI35 shRNA group showed that the expression levels of Beclin-1, LC3-II, and ATG-5 were significantly up-regulated in both Ketr-3 and 786-O cell lines (Figure 4A–D). Next, shRNA-treated cells were infected with a GFP-RFP-LC3 adenovirus for 24 h, and the GFP-RFP-LC3 puncta were monitored by immunofluorescence co-localization assay. GFP-RFP-LC3 adenovirus-infected cells showed a remarkable increase in GFP and RFP intensity compared with the control cells (Figure 4E–H), which implied that IFI35 deletion induced autophagosome accumulation. This conception was again confirmed by the knockdown of IFI35 in renal cancer cells. 

**Figure 5:**
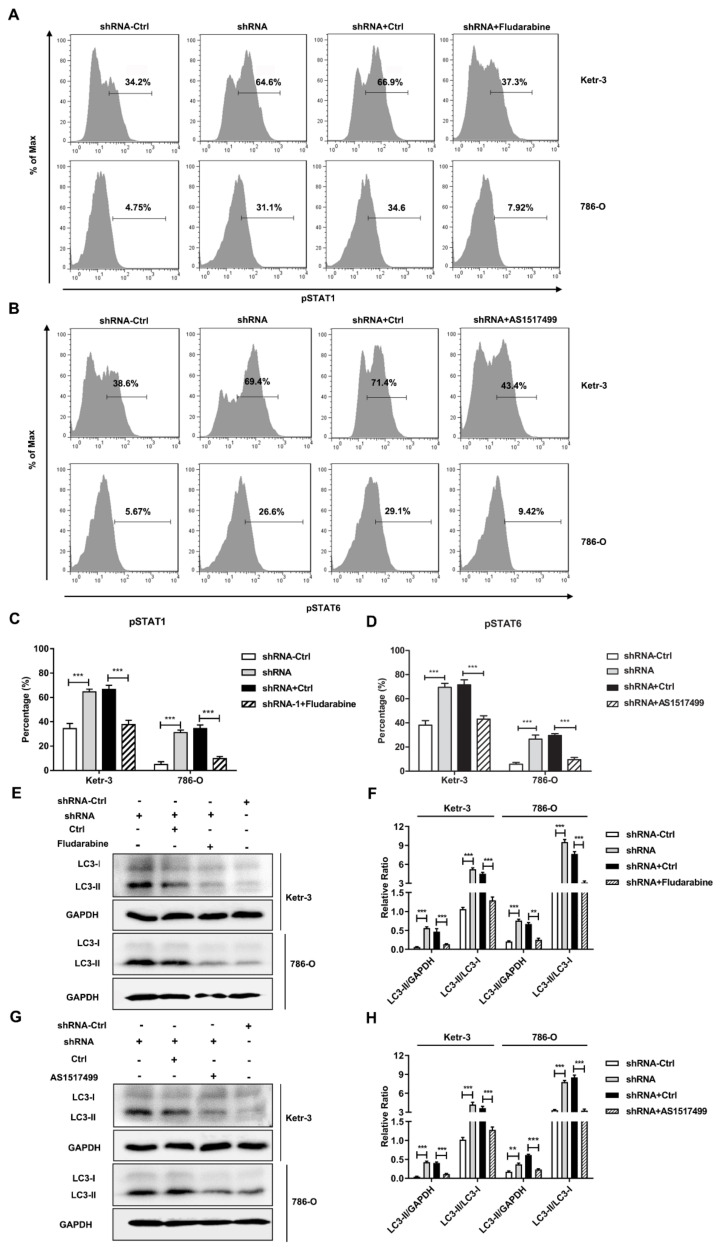
The autophagy induced by the IFI35 knockdown was dependent on the STAT1/STAT6 phosphorylation pathway in renal cancer cells. IFI35 knockdown Ketr-3 or 786-O cells were treated with or without inhibitor fludarabine (5 μmol/L) or AS1517499 (100 nmol/L). (**A**,**B**) Forty-eight hours after inhibitor treatment, the levels of pSTAT1 or pSTAT6 were detected by flow cytometry in IFI35 knockdown renal cancer cells. (**C**,**D**) Statistical analysis of the levels of pSTAT1 or pSTAT6 was represented in (**A**,**B**). (**E**,**G**) Forty-eight hours after inhibitor treatment, the expression of LC3-I and LC3-II were detected by Western blot in IFI35 knockdown renal cancer cells. (**F**,**H**) The panels represent the statistical analysis of the band intensity through densitometric estimation of each band normalized by GAPDH in (**E**,**G**). Data are from one representative experiment of three performed and presented as the mean ± SD, cell samples, *n* ≥ 3. The different significance was set at ** *p* < 0.01, and *** *p* < 0.001. Original blots see Supplementary File S1.

Previous studies demonstrated that STAT1 or STAT6 signaling pathway is involved in autophagy regulation [27,28]. To detect whether these signaling pathways were required for IFI35-mediated autophagy, we first detected phosphorylated expression levels of STAT1/STAT6 in IFI35 shRNA-treated renal cancer cells. The results showed that the levels of pSTAT1 were remarkably increased in IFI35 shRNA-treated Ketr-3 or 786-O cells compared with the control cells (Figure 5A,C). Likewise, pSTAT6 expression levels were also elevated in IFI35 shRNA-treated renal cancer cells (Figure 5B,D). When IFI35 knockdown cells were treated with pSTAT1 or pSTAT6 inhibitors, the expression levels of pSTAT1 or pSTAT6 were suppressed. Notably, pSTAT1 or pSTAT6 inhibitors could remarkably suppress the LC3-II expression and reduced the ratio of LC3-II/LC3-I (Figure 5E–H), implying that IFI35-mediated autophagy was inhibited by the inhibitor in IFI35-deleted renal cancer cells. Taken together, these results indicate that the knockdown of IFI35 could increase the induction of autophagy by the pSTAT1/pSTAT6-dependent pathway in renal cancer cells.

### 3.5. Autophagy Induced by the Knockdown of IFI35 Is Critical for Inhibiting the Malignant Behavior of Renal Cancer Cells and Tumor Growth

**Figure 6:**
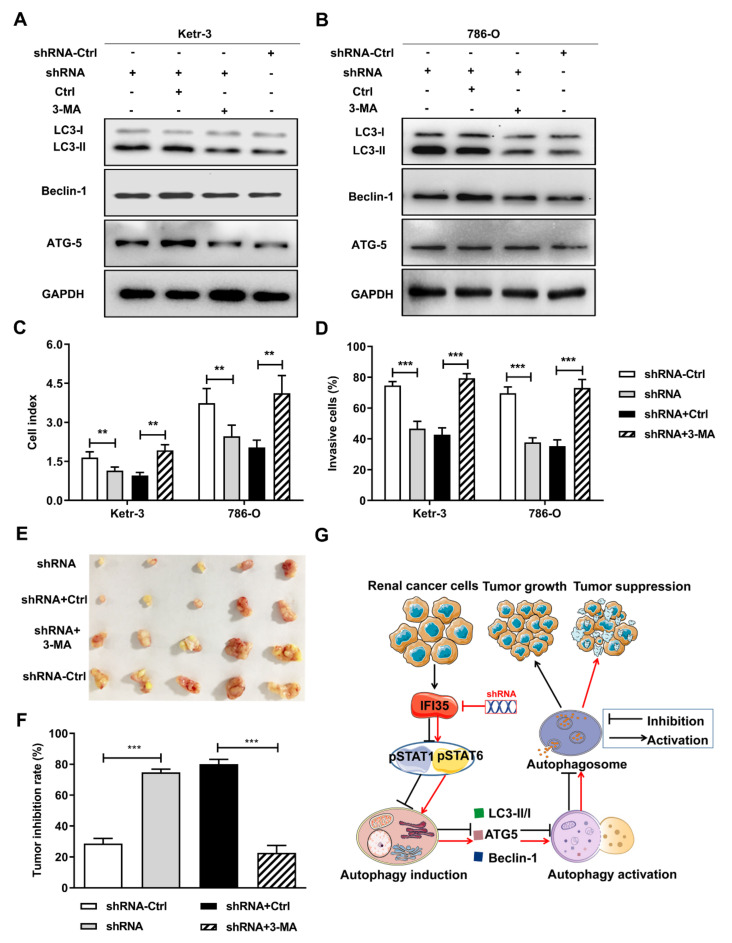
IFI35 knockdown therapeutic efficacy depended on autophagy induction. IFI35 knockdown Ketr-3 or 786-O cells were treated with or without inhibitor 3-MA. (**A**,**B**) Forty-eight hours after 3-MA treatment, the levels of LC3-I, LC3-II, Becline-1, and ATG5 were measured by Western blot in Ketr-3 and 786-O cells. (**C**) RTCA cell proliferation assay was performed in renal cancer cells. (**D**) The invasion of renal cancer cells was detected by the trans-well assay. In 786-O-shRNA cell-xenografted nude mice, the inhibitor 3-MA was administrated intraperitoneally. (**E**) At 45 days after tumor inoculation, the present images of tumor excised from mice were presented. (**F**) Tumor inhibition rate. (**G**) The showed schematic illustration how the knockdown of IFI35 could inhibit renal cancer tumor growth. Knockdown of IFI35 by shRNA in renal cancer cells resulted in the activation of the STAT1/STAT6 phosphorylation pathway, then activated the pSTAT1/pSTAT6-mediated autophagy by up-regulating the autophagy-related gene expression (Beclin-1, LC3-II, and ATG-5) and increasing the ratio of LC3-II/LC3-I, and eventually led to autophagy-mediated tumor inhibition. The experiments were performed with five mice per group. Data are from one representative experiment of three performed and presented as the mean ± SD. Statistical significance was set at ** *p* < 0.01, and *** *p* < 0.001. Original blots see Supplementary File S1.

In order to investigate whether IFI35 regulates the malignant behavior of renal cancer cells by autophagy in RCC, we used an inhibitor 3-MA to block autophagy. Compared with control treatment, 3-MA treatment significantly reduced the expression levels of LC3-II and the rate of LC3-II/LC3-I in IFI35 shRNA-treated Ketr-3 or 786-O cells (Figure 6A,B), implying that the inhibitor could effectively suppress autophagy formation induced by IFI35 knockdown in renal cancer cells. Likewise, the inhibition ability of cell proliferation and invasion by deleting IFI35 were blocked by 3-MA in renal cancer cells (Figure 6C,D). To further evaluate whether the therapeutic efficacy of IFI35 knockdown was dependent on the induction of autophagy in vivo, the subcutaneous 786-O-shRNA tumor models were received intraperitoneal 3-MA. On day 45 after tumor cell inoculation, the suppression of tumor growth by IFI35 shRNA was significantly alleviated in the 3-MA-treated group compared with the control-treated group (Figure 6E). Accordingly, the increased inhibition rate of the tumor was also reversed in the 3-MA-treated group (Figure 6F). Taken together, these results indicated that IFI35 knockdown could inhibit tumor growth by pSTAT1/pSTAT6-dependent autophagy signaling and thus effectively prevent the aggravation of renal cancer (Figure 6G).

## 4. Discussion

RCC is the most common type of renal cancer found in adults and accounts for more than 90% of adult renal carcinomas. It is characterized by high metastasis, poor prognosis, easy relapse, and poor survival, and its pathogenesis is not well understood [7]. For these reasons, we tried to investigate the pathogenesis of RCC in order to find new drug targets for tumor treatment. Many tumor cells escape from cancer immunosurveillance correlates with interferons induce genes (IFNs). As an important interferon inducible gene, IFI35 plays an important role in innate immunity. Therefore, we speculate that it may also play a key role in tumor development. The molecular mechanism of regulation of IFI35- dependent signaling pathway remains unclear. To elucidate the mechanism underlying IFI35 function, we investigated how modifying the IFI35 expression in tumor cell lines and mouse models could influence tumor-inhibition. Through these studies, our results have filled in the gap of knowledge regarding the role of IFI35 in RCC.

Our result showed that the expression of IFI 35 in RCC tissues was significantly higher than that in normal tissues. Correlation analysis showed that the high expression of IFI 35 was significantly related to poor prognosis, but not related to other prognostic indicators such as gender and age. It was not possible to collect normal control of the same individual for 200 RCC patients concurrently, so the expression of IFI 35 was only limited to these samples for research. In addition, IFI35 was an immunoregulatory factor that was also detected in tumor infiltrating immune cells. Therefore, the expression of IFI35 in these immune cells cannot be removed in this study. It is well-known that tumor cells are the main component of tumor tissue, and these cell numbers were far more than the immune cells. Therefore, the expression of IFI35 in infiltrating immune cells was less likely to interfere with the result detected for IFI35 expression in tumor tissues.

The mortality of RCC patients is mainly due to tumor cell metastasis, which was mediated by tumor cell proliferation, migration, and invasion in tumor progression [29,30]. Tumor promoting genes usually are expressed at high levels in cancers and enhance the malignant behavior of tumor cells [31,32]. Our results showed that IFI35 was a tumor-promoting gene. In fact, it inhibited proliferation, migration, and invasion of renal cancer cells treated with IFI35 shRNA compared with the control cells. In addition, the function of IFI35 was further evaluated in the subcutaneous or metastatic renal cancer model. Likewise, the knockdown of IFI35 also suppressed tumor growth or tumor lung metastasis in these two models. These results indicate that IFI35 is a potential therapeutic target for renal cancer.

Autophagy has been reported to be either inhibiting or promoting tumor progression by changing the malignant behavior of tumor cells, implying it is a very important mechanism for cell invasion and metastasis, angiogenesis, or tumorigenesis. In other words, it is suggested that lack of autophagy in tumor cells causes tumor suppression or tumorigenesis through different mechanisms [11,33,34]. Interferons (IFNs) induce the expression of interferon-stimulated genes (ISGs) and play a critical role in innate immune response against numerous viral infections by inhibiting autophagy [35,36]. However, IFI35 autophagy pathway regulation in tumor development is unclear. In this study, in order to investigate the signaling pathway regulated by IFI35, 3-MA, fludarabine or AS1517499 were used to block the autophagy or pSTAT1/pSTAT6 signaling pathway. The use of a cellular inhibitor is an essential way for selecting to bind their target protein, and can keep maintaining homeostasis in the cell, but not damage the cell metabolism. The use of these inhibitors ensured the physiological activity of cells without affecting the authenticity of experimental results.

Our results showed that the knockdown of IFI35 increased the expression of autophagy-related genes (Beclin-1, LC3-II, and ATG-5) in renal cancer cells. We found that autophagic flux was significantly up-regulated in both cell lines. LC3 is divided into the two forms of LC3-I and LC3-II, of which LC3-I is localized in the cytoplasm and LC3-II binds to autophagosomes [37]. Since autophagy converts LC3-I to LC3-II, the increase in LC3-II is used to measure autophagy induction [38]. Our results indicated that the knockdown of IFI35 increased the expression of LC3-II and the ratio LC3-II/LC3-I was higher in renal cancer cells. Likewise, a stronger autophagic flux was observed in the IFI35-deleting group as compared to the control group. In treatment with 3-MA inhibitors, the malignant behavior of tumor cells in IFI35-knockdown renal cancer cells was significantly restored. Moreover, the inhibition of tumor growth by knockdown of IFI35 was abolished in the 3-MA-treated group. Therefore, these results indicate that the knockdown of IFI35 inhibited tumor progression by enhancing the induction of autophagy. 

It must be stated that activation of STATs (e.g., STAT 1,3,6), generally through phosphorylation, activates autophagy. While nuclear STAT3 in the nucleus was capable of orchestrating autophagy through regulation of several genes related to autophagy (these included PIK3C3, PIK3R1, HIF1A, and miRNAs), cytoplasmic STAT3 constitutively inhibited autophagy through interaction with FOXO1/3 and EIF2AK2-sequestration or PKR activity [39,40]. Chen et al. observed that silencing JAK/STAT with ruxolitinib inhibitors increased autophagy in podocytes [41]. Therefore, STAT3 signaling may partially influence IFI35-mediated autophagy in renal cancer cells. However, we found that pSTAT1/pSTAT6 expression shows a significant change in IFI35-deleting renal cancer cells, so the project mainly focuses on the activation of pSTAT1/pSTAT6. IFI35 is an interferon-stimulated gene that is involved with STAT1-mediated cellular proliferation in epithelial cells and tumor cells [23,42]. Likewise, STAT1 and STAT6 activation is also found to be closely related to tumor progression [43,44]. In this study, our results showed that the LC3-II/LC3-I ratio was significantly reducedin the pSTAT1 or pSTAT6 inhibitor-treated IFI35-deleted group as compared with the control group. Therefore, the activation of pSTAT1/pSTAT6 by knockdown of IFI35 in renal cancer cells was involved in the induction of autophagy. We reasonably speculated that the induction of autophagy by knockdown of IFI35 was required for activation of pSTAT1/pSTAT6. Yet, as an inflammatory regulator, IFI35 regulates the inflammatory response of cells, but its impact on autophagy induction is not fully understood. 

Furthermore, STATs are known to form heterodimers with different STAT family proteins. Both IL-6 and type I IFN-γ bring STAT1 and STAT3 to form heterodimers. Notably, STAT1/STAT3 forms when STAT3 is expressed at high levels in response to type I IFNs stimulation, indicating STAT3 sequesters STAT1 into heterodimers and suppresses the formation of STAT1 homodimers [45,46]. It has also been reported in the literature that common γ-chain cytokine signaling, which activates STAT5 through phosphorylation, is required for macroautophagy induction during CD4^+^ T-cell activation. Inhibition of the JAK3 signal induces a series of downstream cytokine receptors, which prevented the induction of macroautophagy in activated T cells. The common γ-chain cytokines could induce T-cell macroautophagy, which was also associated with the increased expression levels of LC3 through the post-transcriptional mechanism [47]. Therefore, the signals mediated by general growth factors and common γ-cytokine promote cell growth and play an important role in regulating STAT5-dependent CD4^+^ T-cell macroautophagy. Future research should investigate whether drugs inhibiting growth factors such as IFI35 could cause tumor regression by inducing macroautophagy in these particular cells. Moreover, the role and mechanism of IFI35-mediated inflammation involved in macroautophagy induction should be further investigated in renal cancer progression.

## 5. Conclusions

In conclusion, the expression and function of IFI35 were investigated in patient specimens, cellular models, and animal models in order to understand the pathogenesis of RCC, and knockdown of IFI35 was investigated as a therapeutic approach to treat renal cancer. Our results suggested that IFI35 expression was significantly correlated with the poor prognosis of RCC patients. Reducing IFI35 expression by shRNA, suppressed the tumor growth of renal cancer, by enhancing autophagy induction, suggesting that knockdown of IFI35 provided a protective role against renal cancer. Therefore, targeting IFI35 may be a therapeutic strategy for renal cancer or autophagy-mediated cancers.

## Figures and Tables

**Table 1:** IFI35 staining and clinicopathological characteristics of 200 RCC patients.

Variables	IFI35 Staining			
	Low (%)	High (%)	Total	*p* *
Age				
>56 years	40 (42.4)	68 (57.6)	113	0.761
≤56 years	36 (47.3)	56 (52.7)	87	
Gender				
Male	52 (56.7)	80 (43.1)	132	0.571
Female	24 (54.3)	44 (45.7)	68	
Tumor size				
≤7 cm	56 (54.6)	98 (45.4)	154	0.383
>7 cm	20 (53.8)	26 (46.2)	46	
Histology Grade				
I–III	67 (72.2)	114 (27.8)	181	0.376
IV	9 (54.2)	10 (45.8)	19	
pT status				
pT1–pT3	67 (62.1)	103 (37.9)	187	0.677
pT4	4 (33.3)	8 (66.7)	13	
pN status				
N0	63 (57.0)	109 (43.0)	172	0.639
N1–N3	8 (54.2)	11 (45.8)	19	
pM status				
M0	68 (51.8)	98 (49.2)	166	0.135
M	2 (66.7)	9 (33.3)	9	
TNM stage				
I–III	54 (59.6)	76 (41.4)	160	<0.05
IV	1 (75.0)	10 (25.0)	9	

* *p* values are from *X*^2^ test.

## Data Availability

The data presented in this study are available on request from the corresponding author.

## Supplementary Materials

The following supporting information can be downloaded at: https://www.mdpi.com/article/10.3390/cancers14122861/s1, Figure S1: The overall staining intensity of IFI35 in tumor tissue (RCC 1, 2, 3 slides) vs normal (NRT slide). File S1: Original blots.
