# Supplementary material for: IFI35 Promotes Renal Cancer Progression by Inhibiting pSTAT1/pSTAT6-Dependent Autophagy
Source: Cancers (Basel). 2022 Jun 9;14(12):2861. doi: 10.3390/cancers14122861 (PMC9221357; doi:10.3390/cancers14122861)
Supplement: Supplementary file 1 [file cancers-14-02861-s001.zip › cancers-1652735-supplementary.pdf]

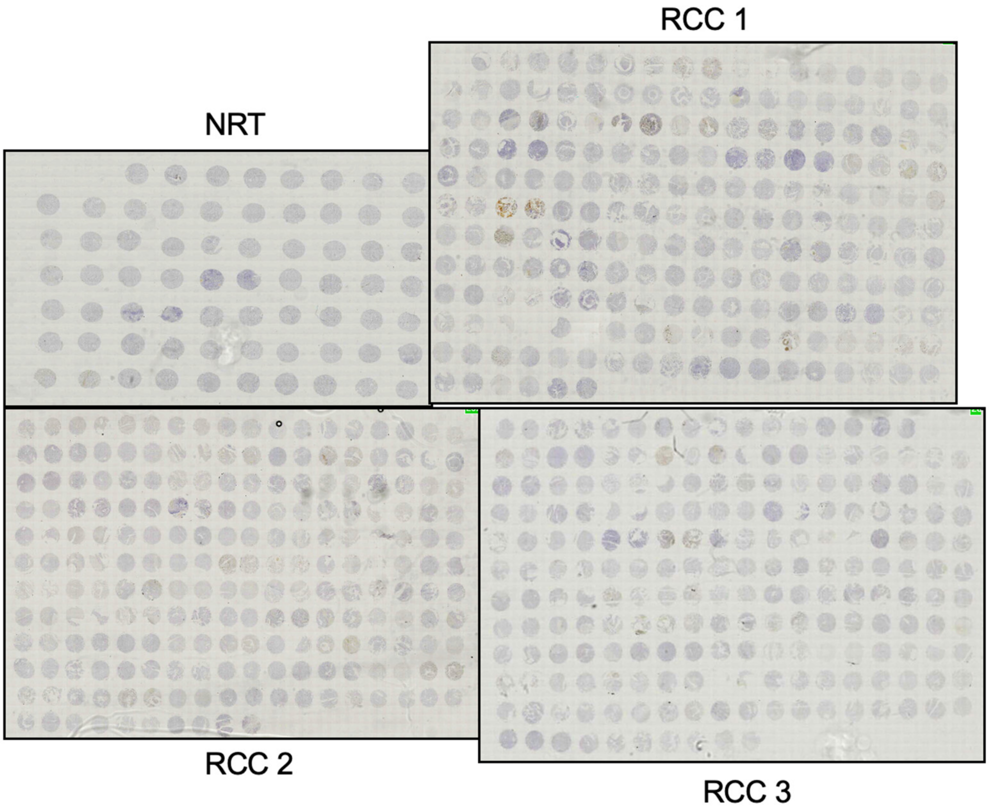

Figure S1. The overall staining intensity of IFI35 in tumor tissue (RCC 1, 2, 3 slides) vs normal (NRT slide).

Supplemental Materials File S1
For the corresponding lanes, please refer to Figure 1 submitted

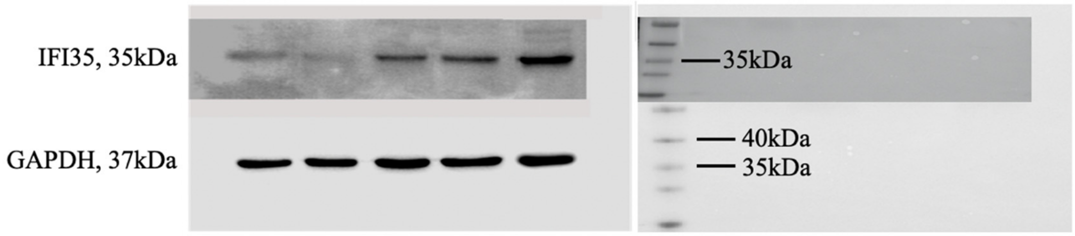

Figure 2A
For the corresponding lanes, please refer to Fig.2 submitted

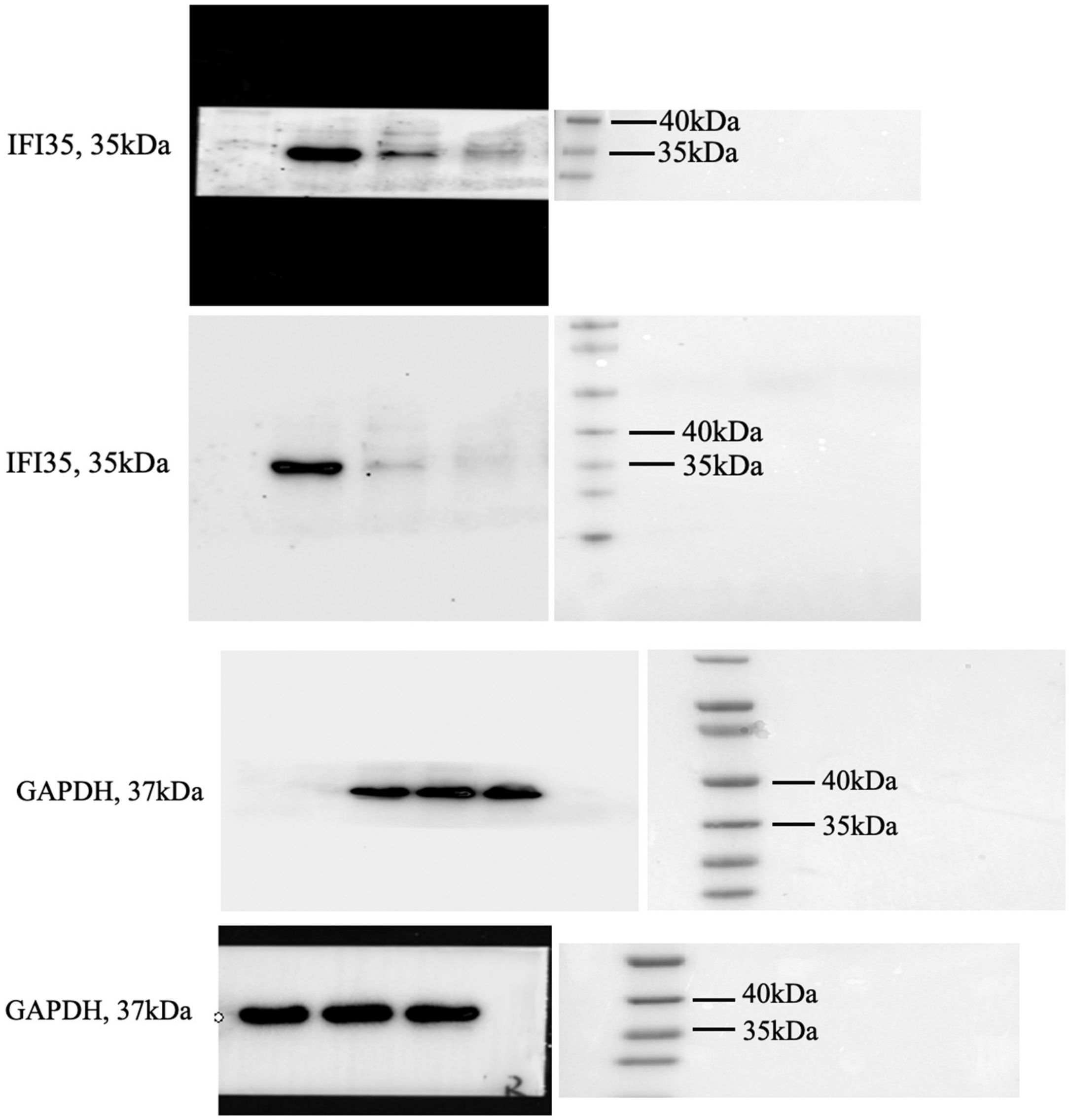

Figure 4A
For the corresponding lanes, please refer to Figure 4 submitted

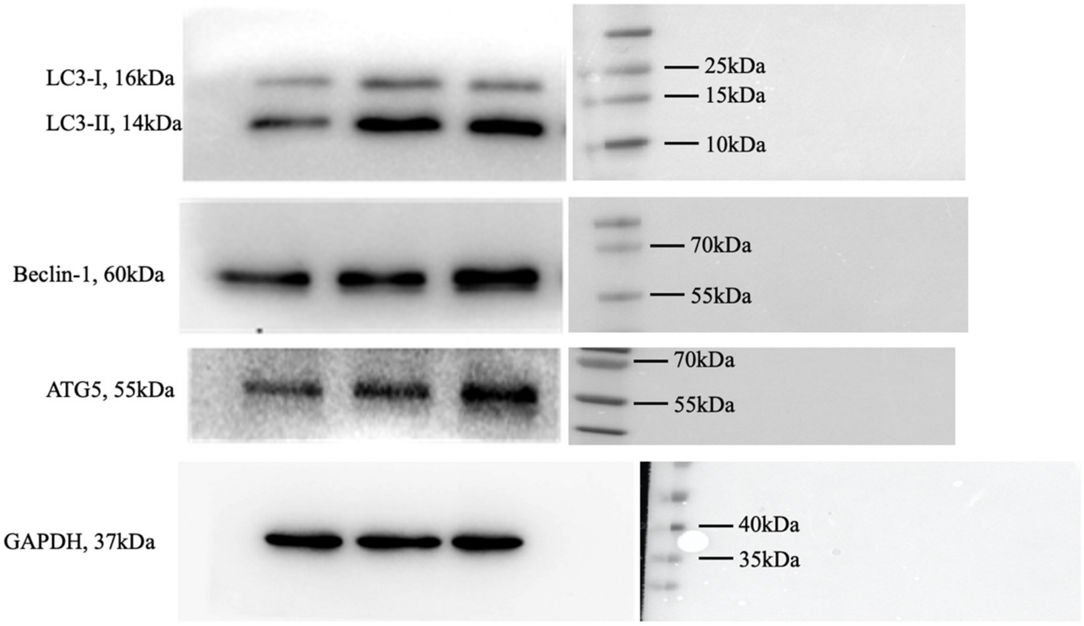

Figure 4C
For the corresponding lanes, please refer to Figure 4 submitted

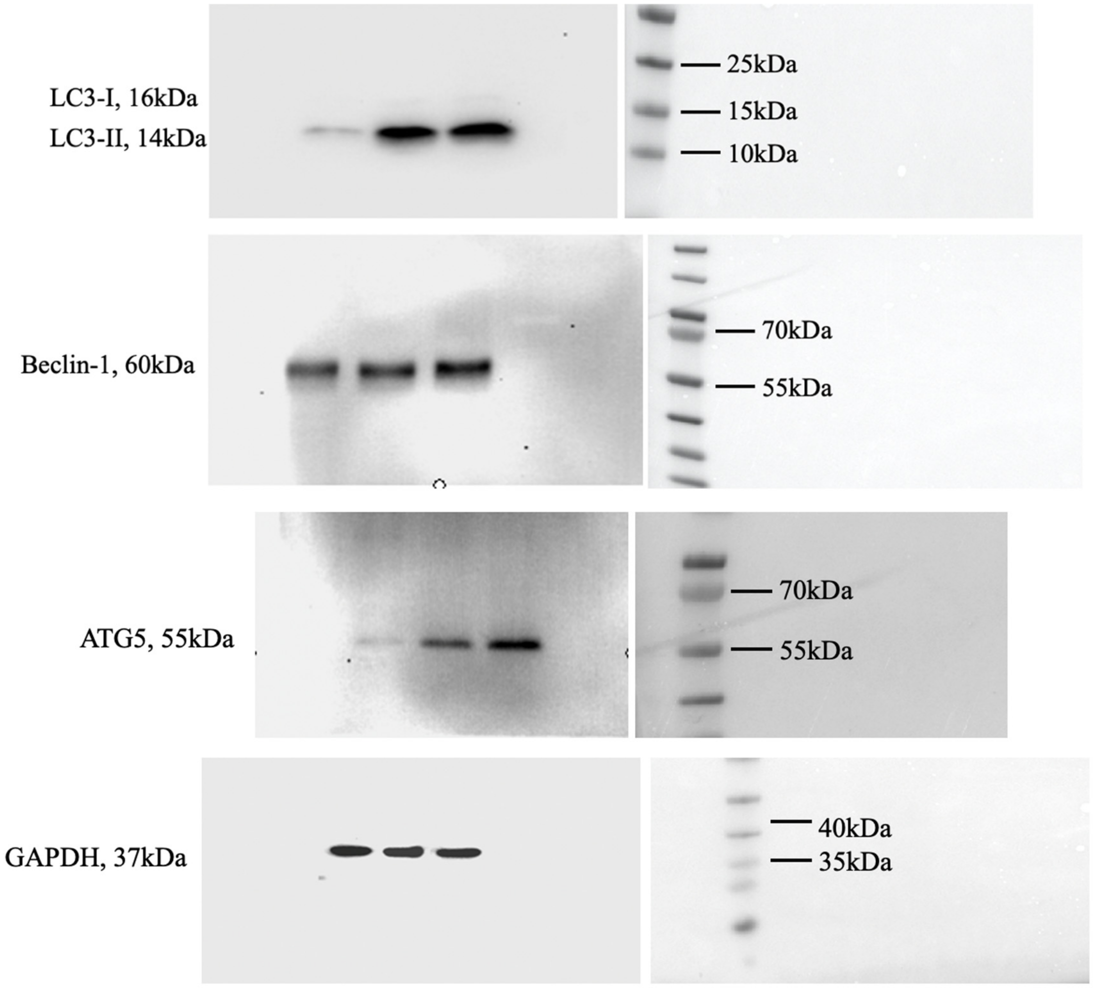

Figure 5E
For the corresponding lanes, please refer to Figure 5 submitted

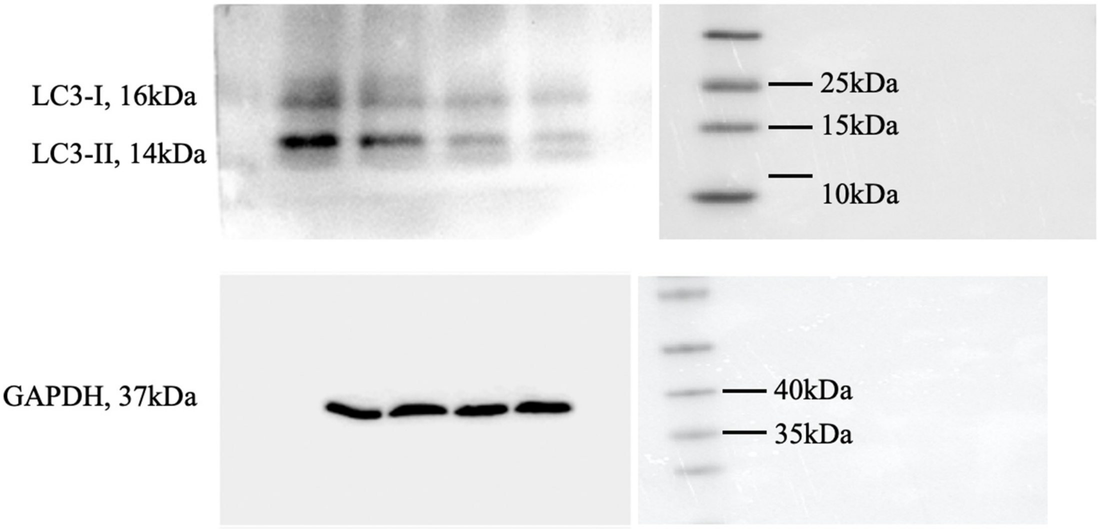

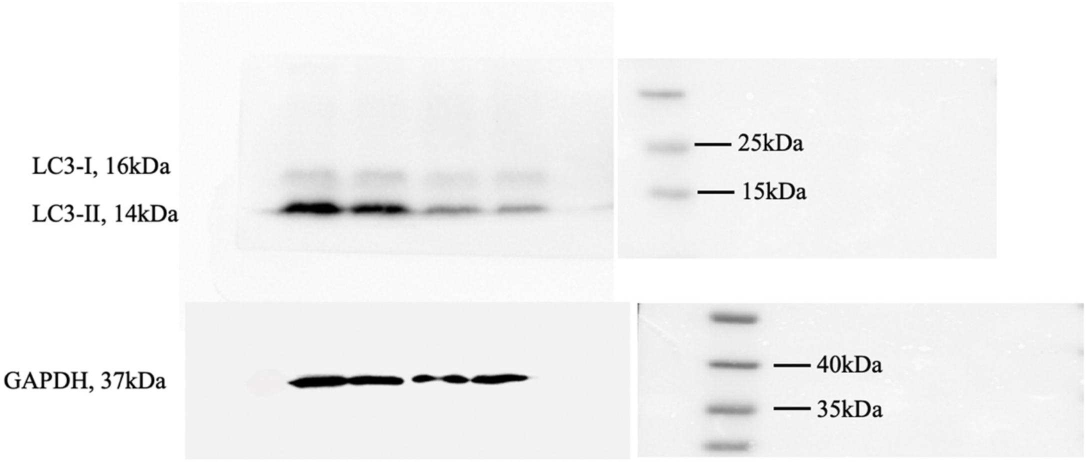

Figure 5G
For the corresponding lanes, please refer to Figure 5 submitted

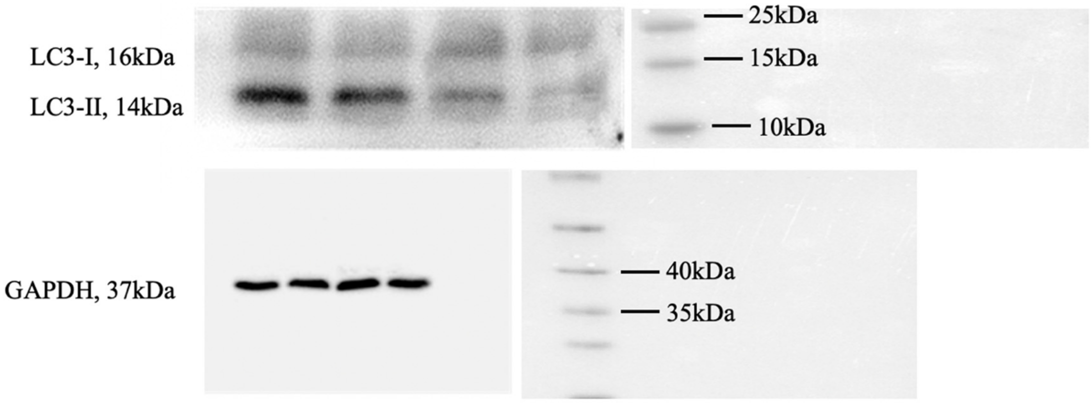

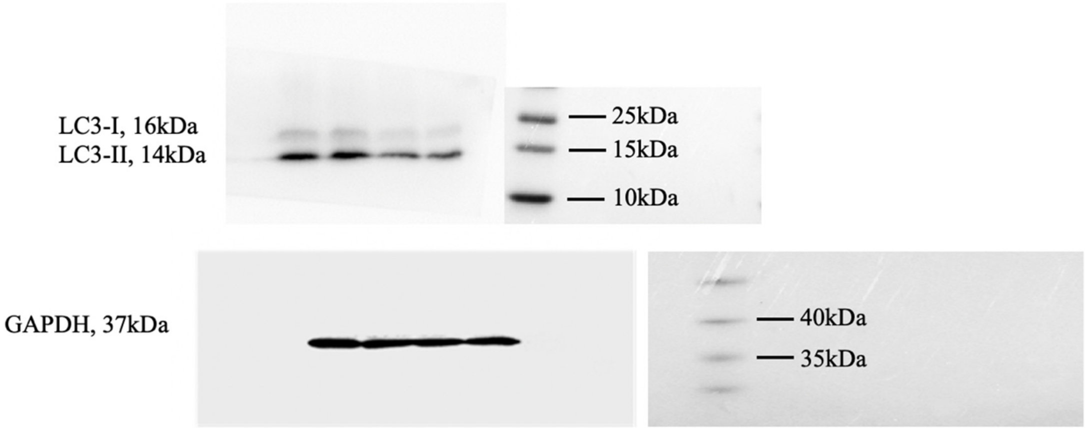

Figure 6A
For the corresponding lanes, please refer to Figure 6 submitted

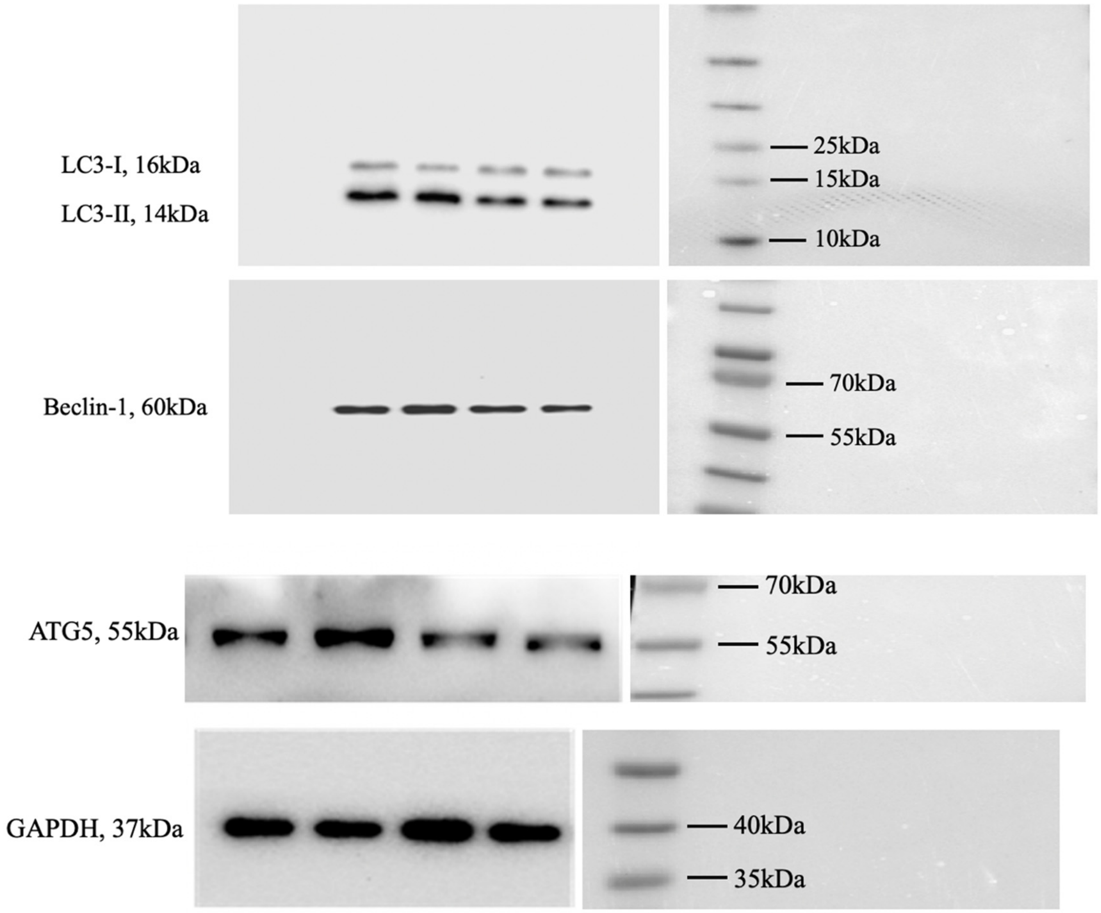

Figure 6B
For the corresponding lanes, please refer to Figure 6 submitted

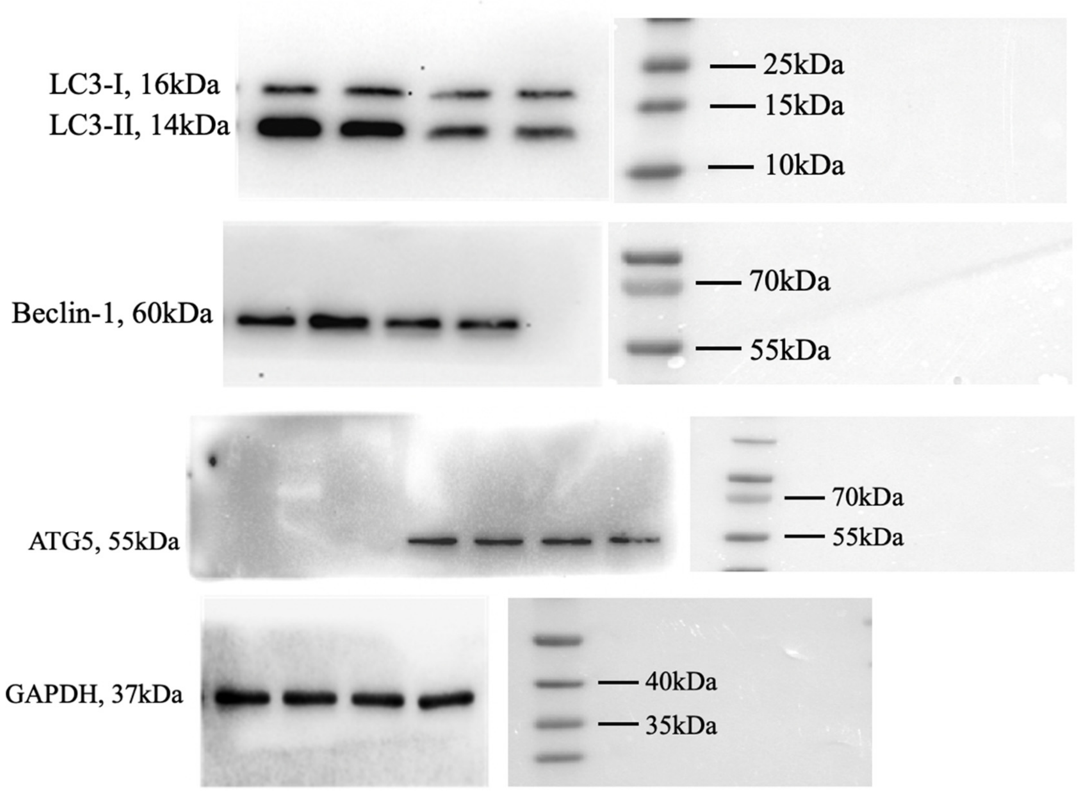
LC3-I, 16kDa
LC3-II, 14kDa
25kDa
15kDa
10kDa
Beclin-1, 60kDa
70kDa
55kDa
ATG5, 55kDa
70kDa
55kDa
GAPDH, 37kDa
40kDa
35kDa
